# Symptomatic Dengue Infection during Pregnancy and Infant Outcomes: A Retrospective Cohort Study

**DOI:** 10.1371/journal.pntd.0003226

**Published:** 2014-10-09

**Authors:** Eleanor E. Friedman, Fadi Dallah, Emily W. Harville, Leann Myers, Pierre Buekens, Gerard Breart, Gabriel Carles

**Affiliations:** 1 Department of Epidemiology, Tulane School of Public Health and Tropical Medicine, New Orleans, Louisiana, United States of America; 2 Franck Joly Hospital, St. Laurent du Maroni, French Guiana, France; 3 Department of Biostatistics and Bioinformatics, Tulane School of Public Health and Tropical Medicine, New Orleans Louisiana, United States of America; 4 INSERM, U1153, Paris, France; Universidade de São Paulo, Brazil

## Abstract

**Background:**

Dengue is a mosquito-borne disease that is common in many tropical and subtropical areas. Dengue infections can occur at any age and time in the lifespan, including during pregnancy. Few large scale studies have been conducted to determine the risk of preterm birth (PTB) and low birthweight (LBW) for infants born to women who had symptomatic dengue infection during pregnancy.

**Methodology/Principal Findings:**

This study is a retrospective cohort study using medical records from 1992–2010 from pregnant women who attended a public regional referral hospital in western French Guiana. Exposed pregnancies were those with laboratory confirmed cases of dengue fever during pregnancy. Each of the 86 exposed infants was matched to the three unexposed births that immediately followed them to form a stratum. Conditional logistic regression was used to analyze these matched strata. Three groups were examined: all infants regardless of gestational age, only infants> = 17 weeks of gestational age and their strata, and only infants> = 22 weeks of age and their strata. Odds ratios were adjusted (aOR) for maternal age, maternal ethnicity, maternal gravidity, interpregnancy interval and maternal anemia. There was an increased risk of PTB among women with symptomatic dengue; (aOR all infants: 3.34 (1.13, 9.89), aOR 17 weeks: 1.89 (0.61, 5.87), aOR 22 weeks: 1.41 (0.39, 5.20)) but this risk was only statistically significant when all infants were examined (p value = 0.03). Adjusted results for LBW were similar, with an increased risk in the exposed group (aOR All infants: 2.23 (1.01, 4.90), aOR 17 weeks: 1.67 (0.71, 3.93), aOR 22 weeks: 1.43 (0.56, 3.70)) which was only statistically significant when all infants were examined (p value = 0.05).

**Conclusions/Significance:**

Symptomatic dengue infection during pregnancy may increase the risk of PTB and LBW for infants. More research is needed to confirm these results and to examine the role of dengue fever in miscarriage.

## Author Summary

Previous studies have reported that dengue fever during pregnancy may be related to preterm birth and low birthweight among infants. However, few studies have used an appropriate control group to compare the risk of these outcomes for infants whose mothers had dengue fever to infants whose mothers did not. We designed this study to provide information on the amount of risk (odds ratios) and the stability of this risk (confidence intervals) of being born preterm or with low birthweight to a mother with documented dengue infection during the pregnancy. In this study there was an increased risk among pregnant women with symptomatic dengue to deliver infants who are preterm or low birthweight, but both the amount of risk and the stability of this risk were affected by the inclusion or exclusion of miscarriages (infants born before 22 weeks of gestational age) This suggests that women who are pregnant should take extra precautions to avoid dengue infections during pregnancy, since it may cause an early delivery, or the birth of a small infant.

## Introduction

Dengue is a mosquito-transmitted viral infection that is common in most tropical and sub-tropical areas. Approximately 390 million dengue virus infections occur each year, and about 500,000 of these require hospitalization [1], [2]. Concern regarding women who are pregnant becoming infected with dengue has been heightened in recent years due to an increase in adolescent and adult infections [3], [4]. Currently, it is unclear if dengue infection in a pregnant woman results in serious health consequences for the mother or the child. Previous research has suggested higher proportions of preterm birth and low birth weight in infants born to mothers who had dengue during pregnancy [5]–[7]. However, many of the previous studies had small sample sizes, poor comparison groups, or other methodological problems [6], [8].

The poor birth outcomes of preterm birth and low birthweight are associated with increased morbidity and mortality. By some estimates, 60% of all neonatal deaths occur to infants who are born preterm or low birthweight [9], [10]. Preterm and low birthweight infants are also more likely to suffer from long term health consequences that can place a significant burden on hospitals, education systems, and the individual families of these infants [11]. This study calculates the risk of preterm birth or low birthweight infants in women who had symptomatic dengue infection during pregnancy, using a retrospective cohort study in French Guiana.

## Materials and Methods

### Ethics statement

As this study collected personal information from French citizens, the requirements and recommendations of the Commission Nationale de l'Informatique et des Libertés were followed. The Tulane University Institutional Review Board approved this study as an exempt study for which informed consent was not sought from subjects. Informed consent was not sought for this study as all information was taken from the existing medical record, and data were analyzed anonymously.

This study used medical archive data from the Franck Joly Hospital in St. Laurent du Maroni, French Guiana. French Guiana is an overseas department of France, with endemic and epidemic transmission of all four serotypes of dengue, as well as a high fertility rate [12]–[14]. Medical data from pregnant women who delivered at Frank Joly hospital during the years 1992–2010 were used in this study. Study subjects were defined as belonging to either exposed or unexposed groups. Exposed subjects were pregnant women who had a laboratory confirmed case of symptomatic dengue fever during pregnancy and who subsequently delivered their infants at Franck Joly Hospital. Many of the exposed subjects were included in previous case series describing the clinical effects of dengue fever in pregnant women [15]–[17].

Unexposed subjects were defined as pregnant women who did not have signs or symptoms of dengue during pregnancy, or who received a negative dengue test result if they were febrile. Unexposed subjects were identified either by using the delivery number of the infant (for fetuses≥22 weeks as determined by ultrasound or≥500 grams), or by using the time of miscarriage of the fetus (for infants <22 weeks of gestation and <500 grams). The three deliveries or miscarriages immediately following the birth of an exposed infant were used as the unexposed matches for the exposed infants. During the time period of this study there were different practices for recording miscarriages. After January 1^st^ 1997, miscarriages of≥17 weeks that occurred at the hospital were recorded in the hospital log books, which previously had only contained information on births≥22 weeks of gestational age. Due to differences in recording miscarriages over time, a sensitivity analysis was conducted. Three levels of inclusion were examined: all fetuses and infants regardless of gestational age and their matches, only fetuses and infants≥17 weeks of gestational age and their matches, and only fetuses and infants≥22 weeks of age and their matches.

For the purpose of this study, a confirmed diagnosis of dengue was a symptomatic case of dengue fever accompanied by a positive test result from one of the following methods: IgM detection by ELISA, viral RNA detection via PCR, a positive NS-1 viral antigen test, or a positive viral culture. Test confirmation was conducted by the Pasteur Institute of Guiana, the national reference laboratory for arbovirus infections in French Guiana.

The outcome definitions used in this study were as follows: a preterm birth was one <37 weeks of gestational age, including miscarriages. An infant with low birth weight was one born weighing <2,500 grams, irrespective of gestational age. Definitions of live birth, stillbirth, and miscarriage used in this study were based on the French definitions [18]. A stillbirth was defined as the birth of a dead infant who weighed≥500 grams or was≥22 weeks of gestational age. A miscarriage was defined as the birth of a dead fetus that was <22 weeks of gestational age and weighed <500 grams. None of the miscarriages included in this study were deliberately terminated pregnancies. Gestational age in this study was determined by ultrasound. Ultrasound information was missing in 5 cases, and gestational age was determined by the date of the last menstrual period (LMP) in two cases, by clinician estimate in two cases, and by both in one case.

Information relating to dengue virus infection, gestational age, and birthweight was abstracted from patient medical files and the Obstetrics and Gynecology ward log books as well as information used to adjust for potential confounders included maternal age, maternal ethnicity, maternal gravidity, interpregnancy interval, and maternal anemia. Adjustment variables were chosen based on a review of the literature and on the information available in the medical archives.

Ethnic information was classified by the investigators, based on information contained in the log books and in the patients' medical file. This information was included as previous studies have indicated that infants with African heritage are at increased risks of poor birth outcomes compared to infants of European heritage [19]–[21]. Maternal ethnicity was dichotomized into mothers of African heritage versus mothers of other ethnic backgrounds. Maternal gravidity was collected as a continuous variable and categorized into four groups (1,2–3,4–5,>5). Interpregnancy interval, the time between a prior pregnancy and the current pregnancy, was dichotomized into a short interpregnancy interval (≤18 months between deliveries) versus a longer interpregnancy interval, or no interpregnancy interval due to being primigravid. Maternal age was categorized into four groups (≤20, 20–29, 30–35,≥36) with the reference group consisting of mothers 20–29 years of age. All data were abstracted from the medical records by an obstetrician trained in abstraction, using a standardized manual of procedures. As almost all infants had complete records, complete case analysis was used in all models.

Statistical analysis for this study included univariate, bivariate, and multivariable investigations. Analyses included descriptive statistical measures for individual variables and tests of association for each variable with the outcomes under investigation. Conditional logistic regression was used to model multivariate associations, with each exposed infant and the three unexposed infants following it modeled as a matched stratum. For the outcome of preterm birth, only dengue infections occurring until the 37^th^ week of gestation were considered. All statistical analyses were done using SAS version 9.2 (Cary, North Carolina).

## Results

A total of 86 exposed infant records were eligible for use in this study and were included in analysis. A total of 281 unexposed infant records were identified as matches to the exposed births by delivery number or time of birth. Out of the 281 records of unexposed infants,,258 were able to be located in the medical archives, and were matched to the exposed infants. One unexposed infant had information missing for several variables, and was not included in multivariate models. Maternal socio-demographic characteristics in the total study sample reflected the larger population of St. Laurent du Maroni, although differences between the exposed and unexposed groups were seen [13], [14], [22]. The exposed mothers were more likely to be of non-African heritage as compared to the unexposed mothers (Table 1). Exposed mothers were also more likely to be anemic and to require a cesarean delivery (Table 1). The exposed and unexposed mothers had similar age distributions, with a mean age of 26.6 years.

**Table 1:** Maternal demographic characteristics among the study population.

	Exposed	Unexposed	
	Number (percent)	Number (percent)	Wald χ^2^
Maternal characteristics	(N = 86)	(N = 258)	P-value
**Maternal ethnicity**			
Amerindian	4 (4.7%)	11 (4.3%)	
Brazilian origin	5 (5.8%)	4 (1.6%)	
Creole	9 (5.8%)	15 (10.8%)	
Maroon	48 (55.8%)	203 (78.7%)	0.02
European	14 (16.3%)	22 (8.6%)	
Other	5 (5.8%)	22 (8.6%)	
Unknown	0 (0%)	1 (1.2%)	
**Maternal age**			
<20	14 (16.3%)	51 (19.8%)	
20–29	39 (45.4%)	119 (46.1%)	
30–35	20 (23.3%)	54 (20.9%)	0.87
36+	13 (15.1%)	34 (13.2%)	
**Maternal Gravidity**			
1	22 (25.6%)	48 (18.6%)	
2–3	29 (33.72%)	75 (29.1%)	
4–5	16 (18.6%)	54 (21.0%)	0.26
6+	19 (22.1%)	81 (31.4%)	
**Pregnancy interval**			
Previous pregnancy loss/termination	6 (7.0%)	9 (3.5%)	
First pregnancy	22 (25.6%)	48 (18.6%)	
1–6 months	4 (4.7%)	23 (8.9%)	0.10
7–18 months	18 (20.9%)	79 (30.6%)	
More than 18 months	36 (41.9%)	99 (38.4%)	
**Anemia**			
No	32 (37.2%)	137 (53.10%)	
Yes	54 (62.79%)	121 (46.9%)	0.01
**Mode of delivery**			
Vaginal	61 (70.9)	212 (82.2%)	
Vaginal with assistance	5 (5.8%)	9 (3.5%)	0.09
Cesarean	20 (23.3%)	36 (14.0%)	
Unknown	0 (0.0%)	1 (0.4%)	

Exposed: fetuses or infants whose mothers had confirmed symptomatic dengue infection during pregnancy.

Unexposed: fetuses or infants whose mothers did not have confirmed symptomatic dengue infection during pregnancy.

Out of the 344 infants included in this sample, 10.5% were born preterm, 13.4% were low birthweight, and 3.8% were stillbirths. Stillbirths were more common among exposed pregnancies than among unexposed pregnancies, regardless of the inclusion or exclusion of miscarriages in the subject population (Table 2). Of the 53 total fetuses or infants who had one or more poor birth outcomes, 54.7% were both preterm and low birthweight, 32.1% were born low birthweight but were not preterm, and 13.2% were preterm but not low birthweight. All of these poor birth outcomes were more common among the exposed fetuses and infants (Table 2).

**Table 2:** Poor birth outcomes among study infants, stratified by maternal dengue fever.

	All infants (N = 344)			Infants≥17 weeks (N = 332)			Infants≥22 weeks (N = 320)		
	Exposed	Unexposed	Wald χ^2^	Exposed	Unexposed	Wald χ^2^	Exposed	Unexposed	Wald χ^2^
Infant outcomes	Number (percent)	Number (percent)	P-value	Number (percent)	Number (percent)	P-value	Number (percent)	Number (percent)	P-value
**Preterm** *									
No	57 (82.6%)	251 (91.3%)		57 (86.4%)	244 (91.7%)		56 (88.9%)	237 (92.2%)	
Yes	12 (17.4%)	24 (8.7%)	0.10	9 (13.6%)	22 (8.3%)	0.4	7 (11.1%)	20 (7.8%)	0.6
**Low birthweight**									
No	69 (80.2%)	229 (88.8%)		69 (83.1%)	222 (89.2%)		68 (85.0%)	216 (90.0%)	
Yes	17 (19.8%)	29 (11.2%)	0.04	14 (16.9%	27 (10.8%)	0.1	12 (15.0%)	24 (10.0%)	0.2
**Stillbirth**									
No	77 (89.5%)	254 (98.5%)		77 (92.8%)	246 (98.8%)		76 (95.0%)	238 (99.2%)	
Yes	9 (10.5%)	4 (1.6%)	0.002	6 (7.2%)	3 (1.2%)	0.01	4 (5.0%)	2 (0.8%)	0.04
**Stillbirth** *									
No	61 (88.4%)	270 (98.2%)		61 (92.4%)	262 (98.5%)		60 (95.2%)	254 (98.8%)	
Yes	8 (11.6%)	5 (1.8%)	0.003	5 (7.6%)	4 (1.50)	0.03	3 (5.0%)	3 (1.2%)	0.1

Exposed: fetuses or infants whose mothers had confirmed symptomatic dengue infection during pregnancy.

Unexposed: fetuses or infants whose mothers did not have confirmed symptomatic dengue infection during pregnancy.

*Only considering dengue cases before 37 weeks of gestation.

Among the 86 dengue-exposed pregnancies, 53.5% of dengue infections occurred in the third trimester, 34.9% in the second trimester, and 11.3% in the first trimester (Table 3). The median gestational age at dengue onset was 29.5 weeks, with a range of 7 to 40 weeks of gestational age, and 69 dengue infections before 37 weeks gestation (80.3%). In dengue-exposed pregnancies, mothers were noted as being febrile at the time of delivery in 25.9% percent of cases, and had threatened preterm labor attributed to dengue in 13 cases (Table 3). Dengue tests were ordered for 37 newborns, with positive test results in 5 (13.5%). In the majority of both maternal and congenital confirmatory testing, IgM tests were used, followed by NS-1 tests (Table 3).

**Table 3:** Characteristics of symptomatic maternal and infant dengue infections.

	Number (percent)
Dengue information	(N = 86)
**Trimester of dengue infection** *	
First trimester	10 (11.3%)
Second trimester	30 (34.9%)
Third trimester	46 (53.5%)
**Maternal dengue diagnosis method**	
IgM	55 (64.0%)
PCR	4 (4.7%)
NS-1	18 (20.9%)
Viral culture	7 (8.1%)
Unknown	2 (2.3%)
**Febrile at delivery**	
No	63 (74.1%)
Yes	22 (25.9%)
**Threatened preterm delivery**	
No	53 (61.6%)
Yes, attributed to dengue	13 (14.9%)
Yes, but not attributed to dengue	5 (5.8%)
Unknown	10 (11.6%)
**Infant dengue diagnosis method**	**Of all tested**
	**(N = 37)**
IgM	20 (54.1%)
NS-1	16 (43.2%)
Unknown	1 (2.7%)
**Confirmed dengue in infants**	**Of all tested**
	**(N = 37)**
No	29 (78.4%)
Yes	5 (13.5%)
Unknown	1(2.7%)

*Trimesters are defined as: 1st ≤14 weeks of gestational age, 2nd 15–28 weeks of gestational age, 3rd trimester≥29 weeks of gestational age.

Unadjusted odds ratios for preterm birth resulted in point estimates that showed an increased risk of preterm birth for women who had symptomatic dengue infections during pregnancy. Odds ratio point estimates ranged from 1.92 for all infants regardless of gestational age to 1.28 for models including infants≥22 weeks and their matches (Table 4). However, none of the unadjusted estimates had significant confidence intervals (p values ranged from 0.10 to 0.61) (Table 4). In adjusted models, point estimates ranged from 3.34 for all infants regardless of gestational age to 1.41 for models restricted to infants≥22 weeks of gestational age and their matched strata. The adjusted odds ratio including all infants regardless of gestational age was significant (aOR = 3.34 (1.13, 9.89)) (p value = 0.03) (Table 5).

**Table 4:** Odds ratios for preterm birth among those with and without symptomatic dengue infection.

	Odds Ratio (95%CI)	Odds Ratio (95%CI)	Odds Ratio (95%CI)
	All infants	Infants≥17 weeks	Infants≥22 weeks
PRETERM BIRTH	(N = 343)	(N = 331)	(N = 319)
**Unadjusted Model**			
Dengue exposed	1.92 (0.88, 4.39)	1.51 (0.64, 3.61)	1.28 (0.49, 3.32)
**Adjusted Model**			
Dengue exposed	3.34 (1.13, 9.89)	1.90 (0.614, 5.87)	1.41 (0.38, 5.20)
Anemia	3.44 (1.15, 10.30)	4.73 (1.42, 15.75)	7.59 (1.73, 33.42)
Maternal ethnicity*	0.63 (0.13, 2.94)	0.66 (0.10, 2.98)	1.37 (0.23, 7.98)
Gravid (1)	Reference	Reference	Reference
Gravid (2–3)	4.10 (0.79, 21.23)	2.43 (0.43, 13.67)	2.41 (0.39, 14.90)
Gravid (4–5)	1.72 (0.28, 10.44)	1.10 (0.18, 6.92)	0.611 (0.07, 5.07)
Gravid (>5)	3.41 (0.50, 23.37)	1.29 (0.17, 9.84)	1.19 (0.15, 9.64)
Interpregnancy interval**	1.56 (0.53, 4.57)	2.12 (0.64, 7.00)	2.83 (0.70, 11.50)
Mothers age <20	0.52 (0.11, 2.58)	0.46 (0.09, 2.44)	0.60 (0.10, 3.50)
Mothers age 20–29	Reference	Reference	Reference
Mothers age 30–35	0.39 (0.09, 1.64)	0.19 (0.03, 1.17)	0.13 (0.02, 1.08)
Mothers age 36+	0.45 (0.08, 2.37)	0.37 (0.06, 2.17)	0.30 (0.05, 1.96)

Exposed: fetuses or infants whose mothers had confirmed symptomatic dengue infection during pregnancy.

Unexposed: fetuses or infants whose mothers did not have confirmed symptomatic dengue infection during pregnancy.

*Mothers of African descent as compared to all others.

** No previous pregnancies or>18 months between pregnancies as compared to ≤18 months.

**Table 5:** Odds ratios for low birthweight among those with and without symptomatic dengue infection.

	Odds Ratio (95%CI)	Odds Ratio (95%CI)	Odds Ratio (95%CI)
	All infants	Infants≥17 weeks	Infants≥22 weeks
LOW BIRTHWEIGHT	(N = 343)	(N = 331)	(N = 319)
**Unadjusted Model**			
Dengue exposed	2.06 (1.03, 4.10)	1.72 (0.83, 3.55)	1.62 (0.76, 3.49)
**Adjusted Model**			
Dengue exposed	2.23 (1.01, 4.90)	1.67 (0.71, 3.93)	1.43 (0.56, 3.70)
Anemia	1.27 (0.55, 2.91)	1.49 (0.63, 3.55)	1.71 (0.68, 4.30)
Maternal ethnicity*	0.39 (0.10, 1.54)	0.28 (0.06, 1.30)	0.49 (0.11, 2.26)
Gravid (1)	Reference	Reference	Reference
Gravid (2–3)	1.28 (0.38, 4.33)	1.01 (0.27, 3.75)	0.86 (0.22, 3.36)
Gravid (4–5)	0.95 (0.22, 4.12)	0.78 (0.17, 3.54)	0.53 (0.10, 2.71)
Gravid (>5)	1.18 (0.26, 5.30)	0.71 (0.14, 3.53)	0.64 (0.12, 3.39)
Interpregnancy interval**	1.42 (0.53, 3.81)	1.52 (0.55, 4.25)	1.77 (0.56, 5.59)
Mothers age <20	1.47 (0.44, 4.93)	1.39 (0.40, 4.81)	1.70 (0.47, 6.13)
Mothers age 20–29	Reference	Reference	Reference
Mothers age 30–35	0.59 (0.17, 2.04)	0.41 (0.10, 1.75)	0.37 (0.07, 1.90)
Mothers age 36+	1.25 (0.32, 4.95)	1.13 (0.27, 4.64)	1.20 (0.29, 5.00)

Exposed: fetuses or infants whose mothers had confirmed symptomatic dengue infection during pregnancy.

Unexposed: fetuses or infants whose mothers did not have confirmed symptomatic dengue infection during pregnancy.

*Mothers of African descent as compared to all others.

** No previous pregnancies or>18 months between pregnancies as compared to ≤18 months.

Unadjusted odds ratios for low birthweight births showed point estimates that indicated increased risk for infants whose mothers had symptomatic dengue infection during pregnancy. Unadjusted point estimates ranged from 2.06 for all infants regardless of gestational age to 1.62 for models restricted to infants≥22 weeks of gestational age and their strata. Only the odds ratio including all infants regardless of gestational age reached statistical significance in unadjusted models (p value = 0.04) (Table 5). After adjustment, point estimates once again showed an increased risk of low birthweight. Adjusted point estimates ranged from 2.23 for all infants regardless of gestational age to 1.43 for models restricted to infants≥22 weeks of gestational age and their matches. However, only the estimate for all infants regardless of gestational age was significant (aOR = 2.23 (1.01, 4.90)) (p value = 0.047) (Table 5).

## Discussion

This study found increases in the risk of preterm birth and low birthweight for infants whose mothers had symptomatic dengue during pregnancy. To our knowledge, the present study is the largest and most epidemiologically sophisticated analysis using individual level data to examine the relationship between dengue fever during pregnancy and poor birth outcomes. This study is also among the first to have examined both the magnitude and variation in the risk of poor birth outcomes while adjusting associations for confounders. Previous research on dengue during pregnancy has generally been in the form of case reports and case series, which sometimes have suggested higher numbers of preterm and low birthweight infants among women with dengue during pregnancy [23]–[27] and sometimes have not [28]–[31]. There have also been studies that have utilized ecologic data to investigate the effect of dengue during pregnancy. A recently published study conducted in Cayenne, French Guiana found an increased risk of preterm birth when dengue transmission was occurring locally during the first trimester of pregnancy, but no significant associations were seen between local dengue transmission and low birthweight infants [32].In the present study we detected increased risks for low birthweight as well as preterm birth in infants whose mothers were infected with dengue. The infants in this study who had low birthweight were also likely to be preterm (Pearsons r = 0.68), suggesting that these infants were low birthweight due to a shorter duration of gestation, rather than impaired fetal growth in utero [33], [34].

In the present study, the largest odds ratios and those that had significant confidence intervals were obtained when the outcomes of interest were examined using the entire population of infants. However, the group containing all infants is also subject to diagnostic bias. Miscarriages <17 weeks of gestational age are more likely to be reported among women who were already at the hospital with dengue symptoms when they miscarried. This diagnostic bias is expected to decrease as gestational age increases and as the miscarriages approach the gestational age of 22 weeks, the age at which mandatory reporting of fetal death begins. The differences in results between these categories suggest that the models including all infants and the models including infants≥17 weeks of gestational age were influenced by the inclusion of the 4 miscarriages at <22 weeks of gestation in the dengue-exposed group. In a recent study by Tan et al., women who had miscarriages at <22 weeks of gestation were more likely to have a positive NS-1 or IgM test for dengue [26], suggesting that it is also possible that the different results obtained in this study reflect actual differences in risk between miscarriage and preterm birth in women who had dengue infection during pregnancy.

Symptomatic dengue infection results in a number of physiologic changes, some of which might result in the initiation of early labor. The immune response to dengue could promote preterm birth by inducing placental inflammation and trophoblast apoptosis, production of inflammatory cytokines and chemokines, or fever [35]. Previous research has shown that some of the cytokines and chemokines released during dengue fever, including Il-6, IL-8 and IL-18, are also seen in preterm delivery [21], [36]–[40]. It is also possible that the presence of fever in response to dengue infection could promote early labor, although the evidence linking fetal loss to febrile episodes is mixed [41], [42]. Several mechanisms have been proposed to explain elevated maternal temperature and fetal loss, including heat shock protein interaction causing damage to the placenta or fetus, and stimulation of uterine contractions [43]–[47].

Interestingly, while the overall study population resembled that of St. Laurent du Maroni, the exposed and unexposed groups differed in regard to ethnicity. Women of non-African descent were overrepresented among women who had dengue during pregnancy (82.35% of the exposed). This may indicate that particular populations are more likely to suffer from symptomatic dengue during pregnancy, or may simply indicate a greater willingness or ability of these women to seek medical treatment [48], [49]. While the French medical system provides universal care, there are transportation barriers to accessing care in this part of French Guiana.

The data used in this study were limited by several constraints. All information used in this study was abstracted from the existing medical record in the archives of the Franck Joly Hospital and was limited by the accuracy and completeness of the medical records. Most importantly, this study was limited by the number of dengue fever cases in the medical record, leading to a lower than desired sample size of exposed pregnancies. While there were few missing data among the variables used in this study, there were other possible confounders that were not examined due to complete absence of the data, in particular, maternal education or socioeconomic status and maternal housing. The time period of this study (1992–2010) encompasses a period with great changes in dengue diagnosis methods. It is difficult to predict how this may have impacted the ascertainment of exposure over time, as all of the diagnosis methods that were used to determine exposure (IgM, viral RNA, NS-1 viral antigen test, and positive viral culture) have benefits and detriments [50], [51]. It is also the case that obstetrical practice has changed during this time period, and changes in the management of complications may have had an impact on the frequency of the outcomes of interest.

This study considered infants as exposed only if their mothers had a symptomatic dengue infection during pregnancy. A large proportion of all dengue infections are asymptomatic, with as many as 90% of all dengue infections occurring without symptoms [52]–[55]. Due to the retrospective nature of this study, we were not able to examine the effect of asymptomatic dengue infection on poor birth outcomes. As testing for dengue was only done if clinically indicated, it is possible that the infants of women with asymptomatic dengue were mistakenly included in the unexposed group. The inclusion of asymptomatic dengue infections in the unexposed group would have either had no effect on our odds ratios, or would have biased our odds ratios towards the null, depending on whether misclassified infants experienced poor birth outcomes.

Despite these limitations, the findings of the present study have great clinical significance for areas with dengue transmission. If dengue infection during pregnancy increases the risk of preterm birth and low birthweight by 40%, then implementation of mosquito avoidance measures during pregnancy should help to lower the risk. The Centers for Disease Control already recommends that pregnant women stay indoors during peak mosquito activity, wear protective clothing, and use insect repellant on clothing and sparingly on skin [56]–[58]. Additional modifications, including screen installation and removal of standing water can be used to reduce the transmission of dengue from *Aedes* mosquitoes to pregnant women in areas where dengue is endemic [59].

Findings from this study also suggest several possible areas of future research. A larger study examining the outcomes of preterm birth and low birthweight is necessary in order to confirm the results of the present study and to allow for more precise confidence intervals. Additional research on the effects of asymptomatic dengue infection on poor birth outcomes, as well as more research on the possible biologic mechanisms linking preterm labor and dengue, are needed i to clarify the relationship between dengue and poor birth outcomes.

## Supporting Information

Checklist S1 STROBE Checklist.
(DOC)
