# Supplementary material for: Symptomatic Dengue Infection during Pregnancy and Infant Outcomes: A Retrospective Cohort Study
Source: PLoS Negl Trop Dis. 2014 Oct 9;8(10):e3226. doi: 10.1371/journal.pntd.0003226 (PMC4191958; doi:10.1371/journal.pntd.0003226)
Supplement: Checklist S1 — STROBE Checklist. (DOC) [file pntd.0003226.s001.doc]

STROBE Statement—Checklist of items that should be included in reports of ***cohort studies***

|  | Item No | Recommendation |
| --- | --- | --- |
| **Title and abstract** | 1 | (*a*) Indicate the study’s design with a commonly used term in the title or the abstract  **Title contains the phrase “retrospective cohort study” (title page)** |
|  |  | (*b*) Provide in the abstract an informative and balanced summary of what was done and what was found  **Abstract contains a summary of methods, resulting odds ratios, confidence intervals, and discussion of significance (page 2)** |
| Introduction | | |
| Background/rationale | 2 | Explain the scientific background and rationale for the investigation being reported  **Previous studies indicating the possible association between dengue and preterm birth/ low birthweight are included in the introduction (page 4)** |
| Objectives | 3 | State specific objectives, including any prespecified hypotheses  **Included in Abstract (page 2) and Introduction (page 4)** |
| Methods | | |
| Study design | 4 | Present key elements of study design early in the paper  **Included in Abstract (page 2) and Materials and Methods section (pages 5-7)** |
| Setting | 5 | Describe the setting, locations, and relevant dates, including periods of recruitment, exposure, follow-up, and data collection  **Included in Materials and Methods section (pages 5-7)** |
| Participants | 6 | (*a*) Give the eligibility criteria, and the sources and methods of selection of participants. Describe methods of follow-up  **Eligibility and selection procedures are described in both the abstract (page 2) and the Materials and Methods section (pages 5-7)** |
|  |  | (*b*) For matched studies, give matching criteria and number of exposed and unexposed  **Matching criteria and number of exposed and unexposed subjected are included in the Abstract (page 2) and Materials and Methods sections (pages 4-5)** |
| Variables | 7 | Clearly define all outcomes, exposures, predictors, potential confounders, and effect modifiers. Give diagnostic criteria, if applicable  **See Materials and Methods section (pages 5-7)** |
| Data sources/ measurement | 8* | For each variable of interest, give sources of data and details of methods of assessment (measurement). Describe comparability of assessment methods if there is more than one group  **See Materials and Methods section (pages 5-7)** |
| Bias | 9 | Describe any efforts to address potential sources of bias  **Confounding factors, as well as changing definitions of miscarriage over time are addressed in Materials and Methods section (pages 5-7)** |
| Study size | 10 | Explain how the study size was arrived at  **See discussion section (page 11)** |
| Quantitative variables | 11 | Explain how quantitative variables were handled in the analyses. If applicable, describe which groupings were chosen and why  **Age was recorded continuously and was categorized for analysis, see Materials and Methods section (page 7)** |
| Statistical methods | 12 | 1. Describe all statistical methods, including those used to control for confounding   **Statistical analyses are covered in the Materials and Methods section (pages 5-7)** |
|  |  | (*b*) Describe any methods used to examine subgroups and interactions  **Not applicable** |
|  |  | (*c*) Explain how missing data were addressed  **Missing data is discussed in the Materials and Methods section (page 7)** |
|  |  | (*d*) If applicable, explain how loss to follow-up was addressed  **Not applicable** |
|  |  | (*e*) Describe any sensitivity analyses  **The sensitivity analysis examining different reported categories of miscarriages is described in the Materials and Methods section (pages 5-7)** |
| Results | | |
| Participants | 13* | (a) Report numbers of individuals at each stage of study—eg numbers potentially eligible, examined for eligibility, confirmed eligible, included in the study, completing follow-up, and analysed  **See Results section (page 8)** |
|  |  | (b) Give reasons for non-participation at each stage  **See Results section (page 8)** |
|  |  | (c) Consider use of a flow diagram  **Not applicable** |
| Descriptive data | 14* | (a) Give characteristics of study participants (eg demographic, clinical, social) and information on exposures and potential confounders  **The characteristics of study population are described in Tables 1-3(pages 17-19) and in the Results section (page 7)** |
|  |  | (b) Indicate number of participants with missing data for each variable of interest  **See Tables 1-5** |
|  |  | (c) Summarise follow-up time (eg, average and total amount)  **Not applicable** |
| Outcome data | 15* | Report numbers of outcome events or summary measures over time  **See Table 1 and Results section (page 8-9)** |
| Main results | 16 | (*a*) Give unadjusted estimates and, if applicable, confounder-adjusted estimates and their precision (eg, 95% confidence interval). Make clear which confounders were adjusted for and why they were included  **See Tables 4 and 5, and Materials and Methods section** |
|  |  | (*b*) Report category boundaries when continuous variables were categorized  **Age, gravidity and interpregnancy interval were continuous variables that were categorized for analysis, see Materials and Methods section (page 7)** |
|  |  | (*c*) If relevant, consider translating estimates of relative risk into absolute risk for a meaningful time period  **Not applicable** |
| Other analyses | 17 | Report other analyses done—eg analyses of subgroups and interactions, and sensitivity analyses **The sensitivity analysis examining different reported categories of miscarriages (pages 6,8)** |
| Discussion | | |
| Key results | 18 | Summarise key results with reference to study objectives  **See Discussion section (page 8)** |
| Limitations | 19 | Discuss limitations of the study, taking into account sources of potential bias or imprecision. Discuss both direction and magnitude of any potential bias  **See Discussion section (pages 9-11)** |
| Interpretation | 20 | Give a cautious overall interpretation of results considering objectives, limitations, multiplicity of analyses, results from similar studies, and other relevant evidence  **See Discussion section (pages 9-13)** |
| Generalisability | 21 | Discuss the generalisability (external validity) of the study results  **See Discussion section (pages 11-12)** |
| Other information | | |
| Funding | 22 | Give the source of funding and the role of the funders for the present study and, if applicable, for the original study on which the present article is based  **See online information** |

*Give information separately for exposed and unexposed groups.

**Note:** An Explanation and Elaboration article discusses each checklist item and gives methodological background and published examples of transparent reporting. The STROBE checklist is best used in conjunction with this article (freely available on the Web sites of PLoS Medicine at http://www.plosmedicine.org/, Annals of Internal Medicine at http://www.annals.org/, and Epidemiology at http://www.epidem.com/). Information on the STROBE Initiative is available at http://www.strobe-statement.org.
